# Immediate-type food allergy to balsam of Peru

**DOI:** 10.1186/2045-7022-1-S1-O39

**Published:** 2011-08-12

**Authors:** Isabel Skypala, Stephen Durham, Guy Scadding

**Affiliations:** 1 Royal Brompton Hospital, London, UK; 2 Imperial College, London, UK

## 

A 48-year old woman presented with a 10-year history of rapid-onset adverse reactions to foods. Reactions involved discomfort in the lips, throat and tongue, throat tightness, an urticarial rash, and a 'sensation of doom'. Suspect triggers included butter toffee popcorn, the aperitif Pernod, cinnamon, toffee apples, ginger nut biscuits, honeycomb confectionary and a supermarket pizza.

Additionally, she described contact hypersensitivity to jewellery and cosmetics. Certain soaps and household detergents also provoked urticaria.

Skin tests and ImmunoCAP tests to aeroallergens and food allergens were negative; prick-prick testing to the same popcorn was also negative. Nonetheless, we felt her history was convincing, and were suspicious of a linking factor between the identified triggers. We subsequently referred her for patch testing.

Patch tests produced a rapid onset urticarial response to myroxylon sulphate (Balsam of Peru); the delayed response was negative. Delayed responses were positive to nickel sulphate and Fragrance mix 1 and 2.

We conclude that our patient had an acute-onset hypersensitivity to Balsam of Peru, an aromatic liquid derived from the Myroxolon balsamum tree used in cosmetics, foods, beverages and medicinal products. It contains cinnamic acid, cinnamyl cinnamate, benzyl benzoate, benzoic acid and vanilla, as well as essential oils similar to those found in citrus fruit peel. It most commonly causes a contact dermatitis; highly allergic individuals may suffer adverse reactions after consuming foods containing Balsam of Peru including soreness of the tongue and mouth. Implicated foods include spices, citrus fruit peel, baked goods, aperitifs, benzoic acid and related preservatives.

Our patient is unusual in presenting with acute symptoms, mimicking IgE-mediated allergy. Of note, contact urticarial reactions have previously been described to patch testing with Balsam of Peru; the mechanism of such reactions is unclear. Clinicians should be aware of non-classical food ‘allergic’ reactions, especially to pre-prepared foods with complex ingredients.

